# Radiation oncology physics coverage during the COVID‐19 pandemic: Successes and lessons learned

**DOI:** 10.1002/acm2.13225

**Published:** 2021-03-20

**Authors:** Nels C. Knutson, James A. Kavanaugh, H Harold Li, Jacqueline E. Zoberi, Tianyu Zhao, Olga Green, Vivian Rodriguez, Baozhou Sun, Francisco J. Reynoso, Alex T. Price, Michael T. Prusator, Taeho Kim, Bin Cai, Geoffrey D. Hugo

**Affiliations:** ^1^ Department of Radiation Oncology Washington University School of Medicine St. Louis MO 63110 USA

## 1 Introduction

The Coronavirus 2019 (COVID‐19) pandemic has severely impacted healthcare systems by putting a massive strain on emergency, intensive care, internal medicine departments, and caregivers. Outpatient care, including radiation oncology, has also been disrupted, forcing departments to modify operations so that patients can continue to obtain treatment in the safest and most effective manner possible. This can be challenging to implement while balancing the goals of continuing to provide high‐quality treatment, complete large‐scale projects, and increase access to care in the community. Throughout this pandemic, our department has adjusted operations by leveraging technology and our diverse skills to ensure that high‐quality patient care could be given while minimizing potential COVID‐19 exposures to patients and staff. During this process, we have learned a great deal from our experience. The goal of this work is to share these lessons so they may be translated across a wide range of medical physics practices.

## 2 Our physics group

Our physics group is organized around six main core values: patient care, integrity, teamwork, community, advancement, and us. These core values help steer the goals of our physics group across all three major branches of service: Clinical, Research, and Education. Our radiation oncology department spans 10 locations, including a large main academic center and nine community‐based practices that range in size from small (single linac) to mid‐size (up to three linacs and brachytherapy). The department as a whole treats approximately 450‐550 patients per day with 100 to 200 patient plans in the planning process. Enterprise services include brachytherapy, external beam therapy, adaptive radiation therapy, quality assurance (QA), proton therapy, treatment planning, stereotactic body radiotherapy, (SBRT), stereotactic radiosurgery (SRS), and satellite operations covered by a team of nearly 40 physicists. The locations of these satellites surround the metro area with three regional satellite locations within a 3‐hour drive. Most of the specialty services (e.g. protons, adaptive) are situated at the main campus, otherwise, the local satellites sites are all integrated into a single, shared external beam service, while the three regional satellites operate largely independently. This structure was designed several years ago to cover all the clinical needs, allow for flexibility in scheduling by having many interchangeable team members. This flexibility is very helpful to ensure robust coverage from week to week; however, it is easy to see this level of mobility could pose a severe risk to the spread of infection between services and locations, as a single physicist could potentially spread the virus very quickly across nearly every location and service.

## 3 Immediate actions

After the first reports of COVID‐19 in the United States, it became clear that our traditional staffing structure had inherent risk and needed modification to minimize interaction and transmission of any infection among staff. If a single physicist could travel between up to five sites in a single week, there was an increased risk of virus transmission between sites. Immediate action was taken to minimize this, with the goal of still maintaining the full range of physics operations needed. A transmission minimization scheme was quickly created and deployed the first week the threat of COVID‐19 in the United States became clear.

Physicists were organized into groups, or “pods”, to cover the different services and locations. All physicists were assigned to a single location and instructed to either work at that location (“on‐site”) or remote work from home (“off‐site”). Each satellite was assigned at least two physicists who alternated coverage on a weekly basis: 1 week on‐site and 1 week off‐site (remote work from home) without overlapping with their partners. At the main campus, each service was treated largely as separate entities to avoid cross‐pod interactions. On‐site assignments were completed on a weekly basis and then rotated to off‐site (home) for at least 1 week. Staffing was limited to only when physically needed in each area, and all other physicists were moved to remote work from home. While working from home, each physicist was expected to remotely apply their particular expertise wherever needed during the day. If a physicist needed to change sites due to a staffing need, a weeklong off‐site coverage was utilized as a buffer when switching between sites. Initially, academic time was limited to only grant‐funded personnel, with all department funded research efforts being temporarily put on hold to better support clinical efforts. This allowed time to carefully assess the pandemic while putting clinical operations at the forefront, and then focus on bringing back education and research operations as deemed safe.

All physicists that were deemed by their physician to be medically at high risk for complications of COVID‐19 were granted an accommodation and worked from home exclusively. The entire dosimetry team was moved to remote work from home. These policy changes coincided with the announcement of stay at home orders in the metro area on March 23, 2020, along with the School of Medicine hold on travel outside of a 60‐mile radius of campus issued on March 18, 2020. To comply with this order, all vacation requests were temporarily revoked and faculty time off was handled on a case‐by‐case basis.

All physicists working on‐site were provided with personal protective equipment (PPE) deemed necessary per hospital policy. All physicists were instructed to practice social distancing, proper hand hygiene, and work area sanitation while on‐site. Employee and patient pre‐screening and mandatory face mask use were implemented across all facilities. To ensure effective communication and coordination of duties under the “pod” and remote work model, a standing 7 am daily COVID‐19 remote huddle was established on March 16, 2020 between the different leaders of the department consisting of the radiation oncology department chair, radiation oncology clinical director, hospital director of radiation oncology, medical physics director, chief of clinical medical physics, department director of business operations, and director of business affairs. Daily remote physics huddles were established to quickly adapt to the ever‐changing situation and disseminate information across all the clinical teams.

## 4 Sustained operations

As the pandemic continued and operations stabilized, daily huddles became weekly, and then as needed based on metro area case load surge and hospital operations. Faculty academic time and educational activities resumed as soon as possible with changes in our standard operating procedures, for example moving teaching to remote classes. Overall, this resulted in a return to a consistent clinical staffing level (i.e., in terms of full‐time equivalent effort) as pre‐pandemic. Areas where we historically thought only on‐site support could occur can now be supported remotely as well as onsite.^1^, ^2^ For instance, one Gamma Knife physicist is on site per our NRC requirements but we can also have a second physicist logged in via remote network keyboard video mouse (IP‐KVM) access and communicating via video conference to provide additional coverage that, pre‐pandemic, would have been on‐site only. Similarly, in brachytherapy, we can supplement on‐site coverage with remote plan checks.

Major projects such as commissioning of a linear accelerator, commissioning of a proton accelerator, and taking on a new regional satellite location were also completed during the pandemic. These were only possible by ensuring our staff stayed safe and healthy and our clinical coverage remained intact. Our staffing model and department culture helped to ensure the success of these challenging projects.

## 5 Major challenges

Staffing and staff safety is obviously a challenge one could face during the pandemic. Even with the best preparations, it is very likely staffing will be affected by direct infection, quarantine due to exposure, or existence of any symptoms identified in the daily screening. Furthermore, these issues are extended to the staff’s family. For example, if a daycare is closed with little notice due to a COVID‐19 exposure and there is no back up childcare option, this can cause major issues in clinical coverage if a parent must take over child care and become unavailable for clinical duty. These unplanned changes in clinical coverage often need to be addressed and communicated out to the team quickly and concisely. This can be challenging and stressful to deal with if there is no built‐in redundancy and backup system in the staffing model. Developing a clear and robust strategy to back fill these unscheduled absences is vital, and without a strong culture of teamwork this can be an immense challenge. Each pod was designed to consist of at least one on‐site physicist and a partner who is rotated off‐site; therefore, a logical first backup is the off‐site physicist with redundant backup coverage from other services second.

Adapting to ever‐changing and uncertain conditions can be extremely frustrating and stressful. When the pandemic started there were many unanswered questions. A year later, some of these have been addressed but many more continue to emerge with regularity. As we learn new information and the action plan changes, effective team communication is crucial, including both dissemination of information from leadership to the team, and within the rest of the group. Keeping in mind these challenges, it is important to deal with the stress and anxiety that can come along with this uncertain and ever‐changing environment. Without strong communication and support from leadership and within the team, this challenge can often feel overwhelming. A morning physics huddle has been a very useful forum to not only share information with the group, but also to get feedback, allow staff to alert leadership to problems, and enable group discussion and consensus on challenging issues. These video calls can help provide a more personal connection and support for each other while safely social distancing.

## 6 Keys to success

A strong work culture has been instrumental in keeping our physics team functioning at a high level in the face of these immense challenges. This has not been easy, but the culture built around our core values is more important than the technology and strategies we have adopted during the pandemic. Our biggest piece of advice to any physics group is to build a strong culture by defining and embracing the core values, that are important to the team, and that will serve as the foundation to rest on.

Effectively designing a remote work from home program was critical to the success of the “pod” model of staffing we implemented, as it helped manage on‐site staffing while providing flexibility in coverage. Crucially, this involved a remote work from home rotation in small teams, ensuring each physicist spent a week of time on‐site every 2 weeks. It is important to remember physicists do much more than QA and checking charts. Physicists are crucial care team members helping to ensure the clinic runs smoothly and safely. Physicists often help coordinate the process and communication amongst the multiple different team members (physicists, physicians, therapists, dosimetrists, etc.) for complicated cases and special procedures. These interactions are extremely valuable to building safe and effective radiation therapy programs. Thus, a remote work from home program for physicists must be carefully designed to ensure physicists are available and present in the clinical environment. For example, naively scheduling all chart check work to be remote and procedural work to be on site would likely result in a similar coverage risk as standard operations, would degrade the team culture, and would likely result in inferior quality.

Employing the pod‐based rotation between on‐site versus remote work has largely been a great success to supplement on‐site coverage while reducing the exposure risk without forcing pure isolation on individuals in the team. The team has embraced numerous remote meeting and communication technologies to bridge the team's physical gap. Leadership supported funds and technology to provide staff with effective remote computing and office tools while providing the necessary resources to ensure that crucial on‐site physics support can continue safely.

To date, not a single case has been cancelled due to a lack of physics availability. Certainly, the pandemic has been challenging for everyone, including our team, both professionally and personally. However, it has also brought the team together to step up to the challenge, strengthening our core values, and cementing our culture. Distributing the clinical workload across a larger group consisting of both on site and remote physicists while building in redundancy, and emphasizing a culture of teamwork, were all key components to our success in maintaining physics operations in the pandemic. Working together to support the entire team allowed us to not only sustain clinical operations but also complete major projects.

The structure of the coverage pods ensures if there is an exposure, it is limited within a relatively small group and not across multiple sites. The pod members are isolated from the rest of the coverage teams, who could then rotate to fill this gap while the exposed group recovers. An exposed pod member also has a “built‐in” minimum quarantine of 5 days due to the week‐on‐week‐off model of coverage. This can easily be extended quickly if the need arises. This structure of working a week on‐site and then a week off‐site numerous times limited the impact of unplanned staffing issues that arose on clinical operations. It is important to note that in addition to social distancing, we implemented “expertise distancing”, meaning the expertise holders remained on separate pods but were still able to effectively share their expertise for an assigned service. For instance, the brachytherapy physics service chief was not scheduled together with the assistant service chief if possible. This was designed to minimize the risk of both being out simultaneously and therefore limiting the available brachytherapy physics resources. Similarly, for staff at our single physicist centers, we paired them with multiple backup physicists who could provide remote support as well as on‐site assistance if the need arose. This is obviously much easier for a large physics group to provide than a solo physicist working independently, but the current pandemic exposes the need for emergency backup coverage.

## 7 Looking ahead

As a group we have been extremely fortunate to have access to the COVID‐19 vaccine. Even though operations are not likely to return to the pre‐pandemic state soon, we are hopeful things will continue to improve over the coming months. From this experience, we see physics groups can continue to be effective in delivering high‐quality and timely patient care while minimizing the risk of virus transmission to staff or patients across a diverse spectrum of radiation oncology practices. The challenges a department of our size faced are no doubt unique, but the actions taken are scalable and can be directly translated to any radiation oncology department. The lessons learned handling a global pandemic can help us and other centers respond effectively to similar emergency situations.
